# Secondary *IDH1* resistance mutations and oncogenic *IDH2* mutations cause acquired resistance to ivosidenib in cholangiocarcinoma

**DOI:** 10.1038/s41698-022-00304-5

**Published:** 2022-09-02

**Authors:** James M. Cleary, Betty Rouaisnel, Antoine Daina, Srivatsan Raghavan, Lauren A. Roller, Brandon M. Huffman, Harshabad Singh, Patrick Y. Wen, Nabeel Bardeesy, Vincent Zoete, Brian M. Wolpin, Julie-Aurore Losman

**Affiliations:** 1 Dana-Farber/Brigham and Women’s Cancer Center, Department of Medical Oncology, Dana-Farber Cancer Institute and Harvard Medical School, Boston, MA USA; 2 SIB Swiss Institute of Bioinformatics, Molecular Modeling Group, Lausanne, Switzerland; 3 Brigham and Women’s Hospital, Department of Radiology, Harvard Medical School, Boston, MA USA; 4 Massachusetts General Hospital Cancer Center, Harvard Medical School, Boston, MA USA; 5 University of Lausanne, Department of Oncology UNIL-CHUV, Ludwig Lausanne Branch, Lausanne, Switzerland; 6 Brigham and Women’s Hospital, Department of Medicine, Division of Hematology, Harvard Medical School, Boston, MA USA

**Keywords:** Bile duct cancer, Cancer therapeutic resistance

## Abstract

The mutant IDH1 inhibitor ivosidenib improves outcomes for patients with *IDH1-*mutated cholangiocarcinoma, but resistance inevitably develops. Mechanisms of resistance and strategies to overcome resistance are poorly understood. Here we describe two patients with *IDH1 R132C*-mutated metastatic cholangiocarcinoma who developed acquired resistance to ivosidenib. After disease progression, one patient developed an oncogenic *IDH2* mutation, and the second patient acquired a secondary *IDH1 D279N* mutation. To characterize the putative *IDH1* resistance mutation, cells expressing the double-mutant were generated. In vitro, IDH1 R132H/D279N produces *(R)*-2HG less efficiently than IDH1 R132H. However, its binding to ivosidenib is impaired and it retains the ability to produce *(R)*-2HG and promote cellular transformation in the presence of ivosidenib. The irreversible mutant IDH1 inhibitor LY3410738 binds and blocks *(R)*-2HG production and cellular transformation by IDH1 R132H/D279N. These resistance mechanisms suggest that *IDH1*-mutated cholangiocarcinomas remain dependent on *(R)*-2HG even after prolonged ivosidenib treatment. Sequential mutant IDH inhibitor therapy should be explored as a strategy to overcome acquired resistance to mutant IDH inhibitors.

## Introduction

Metastatic intrahepatic cholangiocarcinoma (IHCC) is an incurable illness, and until recently, treatment options were limited to cytotoxic chemotherapy^1–3^. Several large-scale genomic profiling efforts have demonstrated the presence of oncogenic isocitrate dehydrogenase 1 (*IDH1*) mutations in approximately 20% of patients with IHCC and oncogenic *IDH2* mutations in approximately 5% of patients with IHCC^2,4–7^. In normal cellular metabolism, IDH1, which is localized to the cytoplasm and peroxisomes, and IDH2, which is localized to the mitochondria, interconvert isocitrate and α-ketoglutarate (α-KG) using NADP(H) as a cofactor^8^. However, cancer-associated mutations in *IDH1* and *IDH2* alter the activity of the mutant enzymes such that they produce the oncometabolite (*R*)-2-hydroxyglutarate (*(R)*-2HG). *(R)*-2HG dysregulates the activities of a number of α-KG-dependent enzymes, including the TET family of DNA demethylases and the Jumonji-domain-containing family of histone lysine demethylases^9^. This results in epigenetic dysregulation, enhanced proliferation, and impaired cellular differentiation. Emerging data suggest that *(R)*-2HG also induces an immunosuppressive tumor microenvironment^8,10–13^. The oral mutant IDH1 inhibitor ivosidenib (AG-120) has demonstrated impressive activity in treating *IDH1*-mutated acute myeloid leukemia (AML), thus providing an important proof of concept for the therapeutic targeting of *IDH1*-mutated malignancies^14^.

Ivosidenib was evaluated in patients with previously-treated metastatic cholangiocarcinoma in the randomized phase 3 ClarIDHy trial^15^. Compared with placebo control, ivosidenib increased progression-free survival, and a prespecified analysis that accounted for crossover from placebo to ivosidenib indicated an overall survival benefit for ivosidenib^15,16^. In contrast to AML, where the overall response rate to ivosidenib is 41%, in IHCC, radiographic partial responses to ivosidenib are rare, and only 2% of ivosidenib-treated patients in the ClarIDHy trial showed an objective radiological response according to RECIST criteria^14,15^. Although ivosidenib is mainly cytostatic in IHCC, a subgroup of patients with *IDH1* mutations on the ClarIDHy trial showed prolonged disease control, and a clinically meaningful increase in the 12-month progression-free survival rate was observed in the ivosidenib versus placebo-treated patients (22% versus 0%)^15^. The results of the ClarIDHy trial led to the approval of ivosidenib for patients with previously-treated *IDH1*-mutated cholangiocarcinoma by the Food and Drug Administration (FDA).

With ivosidenib, as with other targeted therapies, cancer cells inevitably develop resistance. Acquired resistance to ivosidenib has been best studied in AML, where secondary *IDH1* mutations, isoform switching via the acquisition of oncogenic *IDH2* mutations, and the emergence of mutations in *TET2* and the MAPK pathway have been described^17–19^. Data on acquired resistance to ivosidenib in IHCC is limited. One case of conversion of *IDH1 R132C* to *IDH1 R132F*, and one case of isoform switching to mutant *IDH2*, have been reported in patients with *IDH1*-mutated cholangiocarcinoma who developed resistance to ivosidenib^19,20^.

A better understanding of the mechanisms of ivosidenib resistance in *IDH1*-mutated IHCC will help inform the next generation of therapeutic strategies to target mutant IDH1 in cholangiocarcinoma. Here, we describe two patients with *IDH1*-mutated IHCC who experienced long-term stable disease with ivosidenib treatment before ultimately developing resistance. In one patient, disease progression was associated with the acquisition of an oncogenic *IDH2* mutation. The other patient developed a secondary mutation in *IDH1 D279N*. Biochemical, functional, and structural studies show that IDH1 R132H/D279N is resistant to ivosidenib but is sensitive to the novel covalent mutant IDH1 inhibitor LY3410738 and that drug responsiveness correlates with the predicted ability of each drug to bind IDH1 R132H/D279N.

## Results

### Two clinical cases of acquired resistance to ivosidenib

**Fig. 1:**
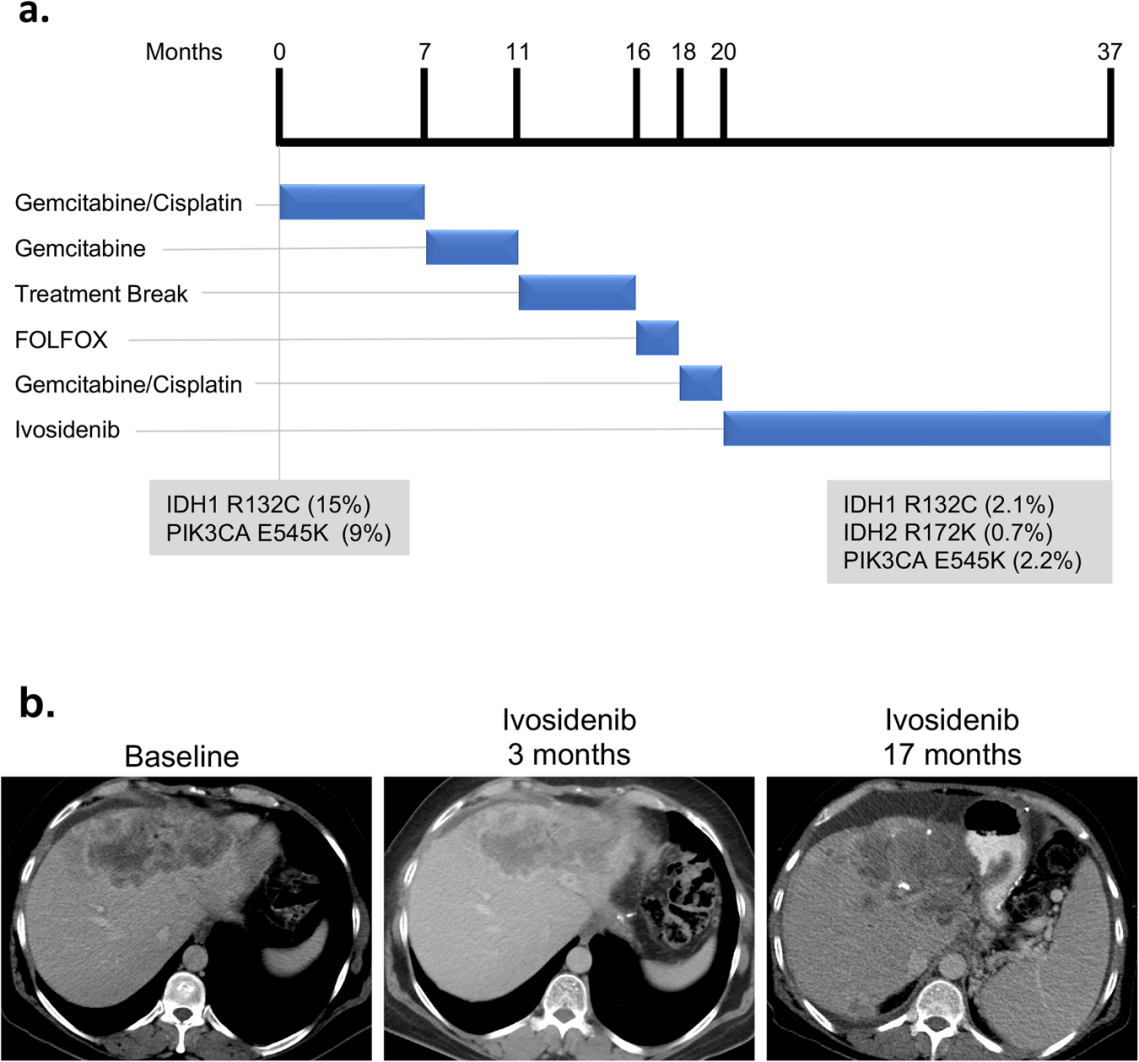
Treatment course for *IDH1*-mutated intrahepatic cholangiocarcinoma—Patient #1. **a** Duration of time on each therapy is shown. Somatic alterations found by sequencing a tumor biopsy (NGS of liver metastasis) at the time of diagnosis and of cfDNA following ivosidenib treatment are indicated below the timeline. Mutation allele frequency (MAF) for each mutation is indicated (%). **b** Computed tomography (CT) scans demonstrating the patient’s cancer at baseline (obtained one day prior to starting ivosidenib) and after 3 and 17 months of ivosidenib treatment.

Patient 1 is a 52-year-old man with a history of a gastric sleeve procedure who initially presented with right subcostal discomfort. A CT scan demonstrated multifocal liver lesions and abdominal lymphadenopathy. A biopsy of one of the liver lesions was consistent with adenocarcinoma of pancreatobiliary origin and he was diagnosed with IHCC. NGS was performed on DNA isolated from his liver biopsy and revealed an *IDH1 R132C* mutation (mutation allele fraction [MAF], 15%) and a *PIK3CA E545K* mutation (MAF, 9%) (Fig. 1a). The patient responded well to first-line gemcitabine/cisplatin chemotherapy. After 7 months of treatment, he transitioned to single agent gemcitabine maintenance therapy for four months and then ultimately took a five-month treatment holiday. Following the treatment break, restaging scans showed progressive disease in his liver and new peritoneal deposits. His cancer rapidly progressed on four cycles of 5-fluorouracil, folinic acid, and oxaliplatin (FOLFOX) chemotherapy. He was then restarted on gemcitabine/cisplatin. His chemotherapy was stopped after 2 months so that he could enroll in the dose expansion of the phase 1 trial of ivosidenib (NCT02073994)^20^. He was treated with ivosidenib 500 mg daily, which he tolerated well with prolonged stable disease (Fig. 1b). After 17 months of ivosidenib treatment, he developed progressive disease in his liver and was taken off the trial. CfDNA analysis performed at the time of disease progression demonstrated persistence of *IDH1 R132C* (MAF, 2.1%) and *PIK3CA E545K* (MAF, 2.2%) and acquisition of a new *IDH2 R172K* mutation (MAF, 0.7%) (Fig. 1a).

**Fig. 2:**
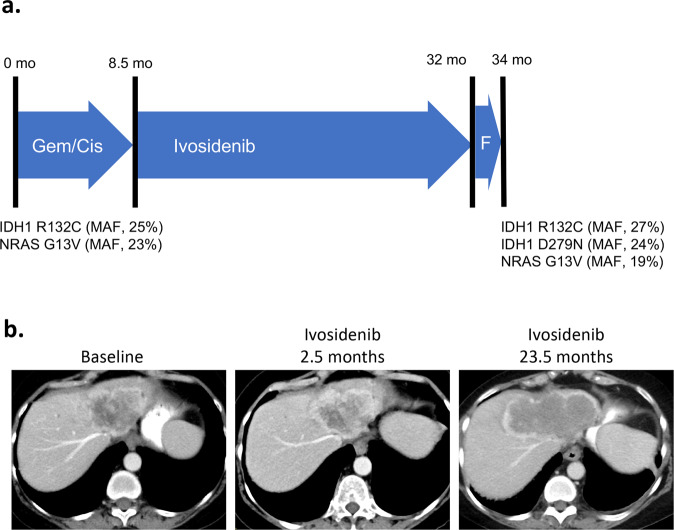
Treatment course for *IDH1*-mutated intrahepatic cholangiocarcinoma—Patient #2. **a** Time of therapy is shown in months (mo). Notable somatic mutations, with mutation allele frequencies (MAF), found by sequencing of a tumor biopsy (liver metastasis) are indicated below the timeline. Gemcitabine/cisplatin (Gem/Cis) and FOLFOX (F). **b** CT scans demonstrating the patient’s cancer at baseline (1 week before starting ivosidenib) and after 2.5 and 23.5 months of ivosidenib treatment.

Patient 2 is a 60-year-old woman with no significant past medical history who initially presented with painless jaundice. Imaging studies revealed an 8.7 cm liver mass with satellite lesions, retroperitoneal lymphadenopathy, and a peritoneal nodule. Biopsy of the liver mass showed adenocarcinoma consistent with a pancreatobiliary primary. Subsequent molecular testing showed *IDH1 R132C* (MAF, 25%) and *NRAS G13V* (MAF, 23%) mutations (Fig. 2a). The patient’s IHCC responded well to first-line gemcitabine/cisplatin for 8.5 months, at which point she developed progressive disease with a metastatic lesion in her acetabulum. After radiation to her acetabulum, she started ivosidenib 500 mg daily. She tolerated ivosidenib well and subsequent scans showed stable disease (Fig. 2b). One year after starting ivosidenib, she developed vision difficulties and was found to have two small brain metastases. She underwent stereotactic radiation to the brain metastases and continued on ivosidenib treatment. Eight months later, she developed progressive disease in her acetabulum and received another course of radiation. She stopped ivosidenib 3.5 months later (after 23.5 months of ivosidenib treatment), when she was found to have radiologic progression of her liver lesions, metastatic lymphadenopathy, and vertebral bone lesions. After disease progression on ivosidenib, she was treated with FOLFOX. However, CT imaging done 2 months later showed that her cancer burden had increased. A percutaneous biopsy of a liver metastasis was performed and NGS analysis demonstrated persistence of the *IDH1 R132C* (MAF, 27%) and *NRAS G13V* (MAF, 19%) mutations. In addition, a novel *IDH1 D279N* mutation (MAF, 24%) was identified (Fig. 2a). After disease progression on FOLFOX, the patient was enrolled in a PD1-directed immunotherapy combination trial, then received off-label treatment with the MEK inhibitor trametinib, and then received off-label treatment with the PARP inhibitor olaparib, but her cancer rapidly progressed on all of these treatments.

### Molecular characterization of *IDH1 R132H/D279N*

**Fig. 3:**
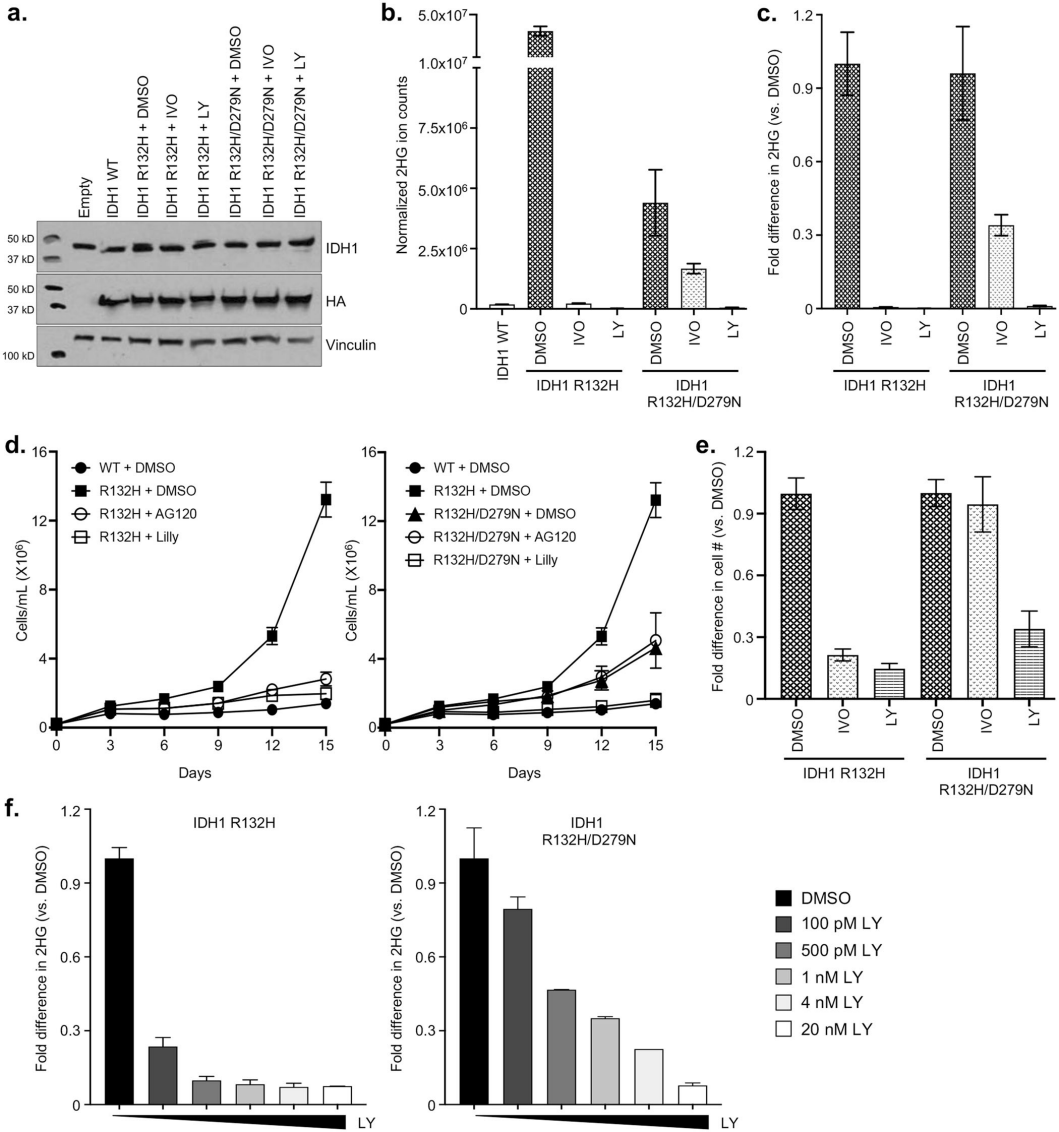
Characterization of IDH1 R132H/D279N. **a** Immunoblot analysis of IDH1 expression in TF-1 cells infected with lentivirus expressing the indicated IDH1 variants and treated with vehicle-control (DMSO), 500 nM ivosidenib (IVO), or 500 nM LY3410738 (LY), as indicated. **b**, **c** Quantification of *(R)*-2HG (**b**) and fold-change in *(R)*-2HG (**c**) in the TF-1 cell lines shown in **a**, as indicated. Shown are mean values (±SD) of duplicate experiments. **d**, **e** Proliferation under cytokine-poor conditions (**d**) and fold-change in day 15 cell counts (**e**) of the TF-1 cells shown in (**a**) The mean (±SD) cell counts of three replicates is shown. **f** Fold-change in intracellular *(R)*-2HG in TF-1 cells expressing IDH1 R132H (left) or IDH1 R132H/D279N (right) treated overnight with the indicated concentrations of LY3410738. Shown are mean values (±SD) of duplicate experiments. In all cases, representative results from at least two independent experiments are shown.

**Fig. 4:**
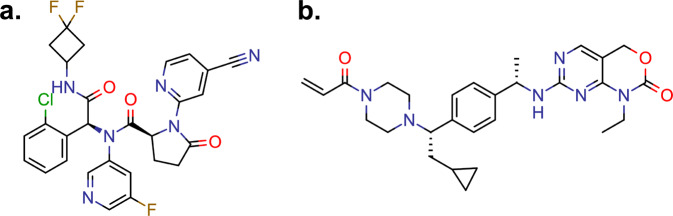
Chemical structures of mutant IDH inhibitors. **a** The non-covalent inhibitor ivosidenib. **b** The covalent inhibitor LY3410738.

We sought to define the biochemical and functional effects of the novel secondary *IDH1 D279N* mutation in vitro using an established mutant IDH transformation assay. In order to assess the ability of the *IDH1* double mutant to both produce (*R*)-2HG and promote cellular transformation, we utilized the TF-1 assay. TF-1 cells are a cytokine-dependent human AML cell line that can be transformed to cytokine independence by (*R*)-2HG^21^. This assay is notable in that the robustness of the cytokine independence induce by (*R*)-2HG is a direct function of intracellular (*R*)-2HG levels. As such, this assay can determine whether a particular level of (*R*)-2HG produced in the presence or absence of drug is sufficient to promote cellular transformation. We expressed wild-type IDH1 (IDH1 WT), IDH1 R132H, or IDH1 R132H/D279N in TF-1 cells and measured *(R)*-2HG levels in the cells by gas chromatography–mass spectrometry (GC–MS). Despite being expressed at similar levels (Fig. 3a), IDH1 R132H/D279N produced ~10-fold less *(R)*-2HG than IDH1 R132H (Fig. 3b). To ask if this level of catalytic activity is oncogenic, we next assessed the ability of IDH1 R132H/D279N to confer cytokine independence to TF-1 cells. Although the transforming activity of IDH1 R132H/D279N was less robust than that of IDH1 R132H, the double mutant was nevertheless capable of inducing TF-1 cytokine independence (Fig. 3d). Ivosidenib potently suppressed *(R)*-2HG production in cells expressing IDH1 R132H but only modestly suppressed *(R)*-2HG production in cells expressing IDH1 R132H/D279N (Fig. 3b, c). Correspondingly, ivosidenib treatment did not affect TF-1 cytokine independence induced by IDH1 R132H/D279N (Fig. 3d, e). Finally, we found that the structurally unrelated mutant IDH1 inhibitor LY3410738^22^ (Fig. 4) potently suppressed both *(R)*-2HG production and cytokine independence in IDH1 R132H/D279N-expressing TF-1 cells (Fig. 3b–f).

### In silico evaluation of ivosidenib and LY3410738 binding to *IDH1 R132H/D279N*

**Fig. 5:**
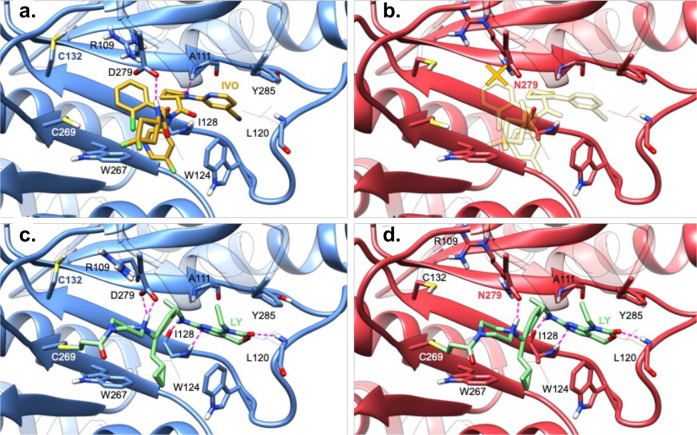
In silico models of mutant IDH1 inhibitors inside the allosteric sites of IDH1 R132C and IDH1 R132C/D279N. **a** Predicted binding mode of ivosidenib (IVO, carbons in yellow) in the allosteric site of IDH1 R132C (carbons in blue). IVO is recognized by IDH1 R132C principally by donating a hydrogen bond to the IDH1 D279 side chain and accepting a hydrogen bond from the IDH1 A111 backbone. Further specific intermolecular interactions involve aromatic contacts with IDH1 W267, IDH1 W124, and IDH1 Y285. **b** IVO is predicted to have impaired binding to IDH1 R132C/D279N (carbons in red) because the chlorophenyl ring clashes with the mutated asparagine side chain (orange X). **c** Predicted binding mode of LY3410738 (LY, carbons in green) in the allosteric site of IDH1 R132C (carbons in blue). LY3410738 covalently binds to IDH1 C269 and displays many favorable electrostatic interactions with the protein: the protonated piperazine makes an ionic interaction with the IDH1 D279 side chain and the inhibitor additionally makes two hydrogen bonds with the IDH1 I128 backbone and accepts a hydrogen bond from the IDH1 L120 backbone. **d** LY3410738 is predicted to adopt a similar covalent binding mode to IDH1 R132C/D279N as it does to IDH1 R132C by keeping all of its numerous anchor points and by replacing the ionic interaction with aspartate with a hydrogen bond to the oxygen of the asparagine-mutated side chain at position 279.

The binding characteristics of ivosidenib and LY3410738 to IDH1 R132C and R132C/D279N were investigated by molecular docking studies that were complemented by molecular mechanics optimization in the IDH1 single-mutant and IDH1 double-mutant modeled structures. Both inhibitors are well accommodated in the IDH1 R132C allosteric site (Fig. 5a, c). They occupy roughly the same space in the cavity and, notably, neither inhibitor comes in contact with IDH1 C132. Molecular recognition of ivosidenib is mainly governed by the donation of a hydrogen bond to the side chain of IDH1 D279 and acceptance of a hydrogen bond from the backbone of IDH1 A111. Other specific interactions are aromatic contacts with IDH1 W267, IDH1 W124, and IDH1 Y285. In contrast, LY3410738 displays more numerous and stronger anchor points: the covalent bond to IDH1 C269, the ionic interaction with IDH1 D279, and three different hydrogen bonds deep in the cavity to the backbones of IDH1 I128 and IDH1 L120. Other favorable interactions include π contacts with IDH1 W124 and IDH1 W267.

The mutation from an acidic aspartate (D) to a neutral asparagine (N) at position 279 induces important changes in the local properties and shape of the allosteric binding site of IDH1 R132C/D279N. Ivosidenib is predicted to have impaired binding to the double mutant (Fig. 5b). The reshaped space in the vicinity of IDH1 N279, along with the change in orientation of IDH1 R109 and the rotation of the asparagine, clash with the fluorophenyl moiety. Molecular docking was not able to generate an appropriate geometry to achieve a hydrogen bond with IDH1 N279 or to replace this critical interaction with another favorable interaction with the protein. In contrast, LY3410738 binding is minimally impacted by the *IDH1 D279N* mutation (Fig. 5d). The intermolecular interaction pattern of LY3410738 within the double-mutant allosteric pocket was calculated to be identical to that of the R132C single-mutant allosteric pocket. The binding mode similarities in IDH1 R132C and in IDH1 R132C/D279N provide a strong structural rationale for the preserved inhibitory activity of LY3410738 against IDH1 R132C/D279N.

## Discussion

Pharmacological inhibition of mutant IDH1 offers a greatly needed therapeutic option for patients with *IDH1*-mutated cholangiocarcinoma, but, as with other targeted therapies, its effectiveness is limited by the development of acquired resistance. The molecular mechanisms leading to acquired resistance to ivosidenib in *IDH1*-mutated IHCC remain poorly understood. Here, we describe two patients with *IDH1*-mutated IHCC who clinically benefitted from ivosidenib for at least 17 months. Mutant IDH isoform switching through the development of an oncogenic *IDH2* mutation was likely responsible for Patient #1’s acquired resistance to ivosidenib. Patient #2 developed a secondary *IDH1*-mutation, *D279N*, which confers resistance to ivosidenib in vitro and likely contributed to her acquired resistance to ivosidenib and her clinical disease progression.

Ivosidenib allosterically inhibits the production of *(R)*-2HG by R132-mutated IDH1 proteins by locking mutant IDH1 in its inactive (open) confirmation^17^. One identified resistance mutation to ivosidenib in AML, *IDH1 S280F*, is hypothesized to sterically block the binding of ivosidenib to its binding site^18,20^. Patient #2 developed a mutation in a neighboring residue, aspartate 279, which could similarly affect the binding kinetics of ivosidenib. The *IDH1 D279N* mutation has previously been reported in AML and was presumed to be a drug-resistance mutation^17^. In agreement with this supposition, we found that cells expressing IDH1 R132H/D279N are resistant to ivosidenib, both biochemically, as determined by their ability to produce *(R)*-2HG in the presence of ivosidenib, and functionally, as determined by their ability to confer cytokine independence to TF-1 cells in the presence of ivosidenib. In silico modeling of the effect of IDH1 D279N on the structure of mutant IDH1 confirmed that the ivosidenib resistance of IDH1 R132H/D279N is due to disruption of drug binding.

The limited therapeutic options for patients with IHCC create a great need for therapeutic strategies that can overcome acquired resistance to ivosidenib. In patients with IHCCs bearing *FGFR2* alterations, the covalent FGFR inhibitor futibatinib can overcome many of the acquired resistance mutations induced by treatment with reversible FGFR inhibitors^23^. In our cell-based assays, the irreversible mutant IDH1 inhibitor LY3410738 was able to potently inhibit *(R)*-2HG production and block transformation mediated by IDH1 R132H/D279N. This was further confirmed by in silico modeling that revealed that IDH1 D279N minimally impacts the binding of LY3410738 to IDH1 R132H/D279N. LY3410738 has recently entered clinical trials for the treatment of *IDH1*-mutant hematologic malignancies (NCT04603001) and *IDH1*-mutant solid tumors (NCT04521686). Determining whether this drug can overcome acquired resistance to ivosidenib is a question of great clinical importance.

The ability of acquired *IDH2* mutations to drive resistance to ivosidenib has been described previously in AML and in a patient with cholangiocarcinoma^17–20^. This phenomenon of isoform switching reinforces the concept that *IDH1*-mutated cancers are specifically dependent on *(R)*-2HG rather than on other possible consequences of *mutant IDH1* acquisition. The mutant IDH2 inhibitor enasidenib is very effective in treating *IDH2*-mutated AML and is FDA-approved for this indication^24^. While the efficacy of enasidenib in cholangiocarcinoma has not yet been evaluated, one possible therapeutic strategy to overcome mutant IDH1/2 isoform switching would be combination therapy with ivosidenib and enasidenib, or treatment with dual mutant IDH1/IDH2 inhibitors^25^.

Despite having a co-occurring *NRAS* codon 13 mutation, Patient #2 showed prolonged clinical benefit with ivosidenib. *NRAS* is mutated in ~3% of cholangiocarcinomas and efforts to therapeutically target *NRAS* mutations in patients have been unsuccessful to date^4,6,26^. *NRAS* mutations promote primary and secondary resistance to ivosidenib in AML but are not a universal marker of resistance, as one study reported a complete response to ivosidenib in 2 of 23 patients with *NRAS*-mutant AML^14,17^. Further studies are needed to define the effects of *NRAS* and other MAPK pathway mutations on primary and acquired resistance to ivosidenib in *IDH1*-mutated cholangiocarcinoma.

In conclusion, targeted therapies directed against mutant IDH1 have opened a new therapeutic avenue for patients with IHCC with *IDH1* mutations, and the FDA recently approved ivosidenib for previously-treated *IDH1*-mutated advanced cholangiocarcinoma. Here, we describe two patients with *IDH1*-mutated IHCC who developed acquired resistance to ivosidenib after the acquisition of an oncogenic *IDH2* mutation or a drug-resistant secondary *IDH1* mutation. In both cases, the mechanisms of acquired resistance suggest that additional therapeutic strategies that target mutant IDH can overcome this resistance. Therapeutic strategies using sequential mutant IDH inhibitors may be feasible and effective and merit further investigation.

## Methods

### Patient data

Patients were enrolled in the dose expansion of the phase 1 trial of ivosidenib in solid tumors (NCT02073994)^20^. OncoPanel next-generation sequencing (NGS) was performed as part of an institutional genomic profiling effort^27^. OncoPanel is a CLIA-certified laboratory assay (CLIA certificate: 22D2040971) that uses hybridization-based capture to detect mutations in 447 cancer-associated genes. Both studies were conducted according to the principles of the Declaration of Helsinki and the International Conference on Harmonization Good Clinical Practice guidelines. The studies were approved by the Dana-Farber/Harvard Cancer Center Institutional Review Board. Cell-free DNA (cfDNA) analysis was performed using a CLIA-approved commercially available assay (Guardant 360)^28^. Informed consent was obtained from all human participants.

### Biochemical and functional characterization of IDH1 mutants

#### Cell lines, cell culture, and drug treatments

HEK-293T cells (ATCC) were maintained in Dulbecco’s Modified Eagle’s Medium (DMEM), containing 10% fetal bovine serum (FBS) and 1% penicillin/streptomycin. TF-1 cells (ATCC) were maintained in RPMI containing 10% FBS, 1% penicillin/streptomycin, and 2 ng/mL recombinant human GM-CSF (R&D Systems). Lentiviral particles were generated by Lipofectamine 2000 (Invitrogen) co-transfection of HEK-293T cells with control or IDH1 expression vectors and the lentiviral packaging constructs psPAX2 (GAG-Pol) and pMD2.G (VSV-G) in a 2:2:1 ratio. Lentiviral infections were performed as previously described in ref. ^21^, and the infected cells were selected with 1 μg/mL puromycin starting 24 h after infection. TF-1 cells were treated with ivosidenib (Selleck Chemicals, LLC) or LY3410738 (Eli Lilly) prior to immunoblot analysis, 2HG measurement, and growth factor deprivation. Cell lines were authenticated by Short Tandem Repeat (STR) profiling. Cell lines repeatedly tested negative for mycoplasma throughout the experimental period.

#### Vectors

The lentiviral expression vector used to express the IDH1 variants (pMT040-IRES-PURO) was custom-made (Genscript). It contains a CMV promoter driving the expression of a single transcript encoding a multiple cloning site, an IRES sequence, and a puromycin resistance gene. A sequence encoding HA-tagged *wild-type IDH1* (Accession# NM_005896) was cloned upstream of the IRES sequence to produce the vector pBT040-HA-IDH1^WT^-IRES-PURO. Point mutations to generate *IDH1 R132H* and *IDH1 R132H/D279N* were introduced into pBT040-HA-IDH1^WT^-IRES-PURO by site-directed mutagenesis (QuikChange II XL, Agilent).

#### Immunoblot analysis

Whole cells extracts were prepared in lysis buffer (50 mM Tris-HCl pH 7.9, 400 mM NaCl, and 0.5% NP40) supplemented with protease inhibitor cocktail (Roche), resolved on 4–20% SDS-PAGE gels (BioRad), and transferred to 0.45 µm PVDF membranes (Millipore). The membranes were blocked in TBST with 5% non-fat milk, probed with primary antibodies, and detected with horseradish-peroxidase-conjugated anti-rabbit (Jackson ImmunoResearch Laboratories, #211-032-171) or anti-mouse (Cell Signaling Technologies, #7076) antibodies. Anti-rabbit secondary antibody was used at a 1:10,000 dilution and an anti-mouse secondary antibody was used at a 1:5000 dilution. Primary antibodies used: rabbit monoclonal HA-tag (Cell Signaling Technology, #3724) at a 1:1000 dilution, rabbit polyclonal anti-IDH1 (Cell Signaling Technology, #3997) at a 1:500 dilution, and mouse monoclonal anti-vinculin (Sigma, V9131) at a 1:1000 dilution. The order of antibody probing was as follows: anti-IDH1, then anti-HA, then anti-vinculin, and, between each probing, the membrane was stripped with Restore PLUS Western Blot Stripping Buffer (Thermo Scientific) and re-blocked with 5% non-fat milk. Uncropped western blots are shown in Supplementary Fig. 1.

#### Gas chromatography–mass spectrometry (GC–MS)

Metabolites were extracted from exponentially growing TF-1 cells using 80% aqueous methanol (−20 °C) and were profiled by GC–MS as previously described in ref. ^21^.

#### TF-1 proliferation assays

Cytokine independent proliferation of TF-1 cells was assessed as previously described in ref. ^21^. In brief, after lentiviral infection, cells were passaged for 8 days in GM-CSF-rich media supplemented with puromycin. Cells were then passaged for an additional 2 weeks in GM-CSF-rich media supplemented with puromycin and either DMSO or mutant IDH1 inhibitor. The cells were then washed four times with plain RPMI and 2 × 10^5^ cells/mL were plated in triplicate in media lacking GM-CSF supplemented with vehicle-control (DMSO) or mutant IDH1 inhibitors ivosidenib or LY3410738^29,30^. Cell proliferation was assessed by counting the number of viable cells/mL every 3 days using a Vi-Cell Cell Viability Analyzer (Beckman Coulter).

### Structural characterization of IDH1 mutants

#### Computational structural modeling

Both IDH1 R132C and IDH1 R132C/D279N in silico models were built from the crystal structure of IDH1 R132H in complex with a small molecule allosteric inhibitor (PDB:6U4J, chain A). Virtual mutations of side chains at positions 132 and 279 followed by local optimization in the Amber Force Field were performed in the UCSF Chimera environment (version 1.15, www.cgl.ucsf.edu/chimera/)^31,32^. Both structures were prepared by removing crystallographic water and ligand molecules, adding hydrogens, and computing Gasteiger atomic charges^33^. Modeled IDH1 R132C and IDH1 R132C/D279N tridimensional structures were considered as docking targets for ivosidenib and LY3410738. The chemical structure of ivosidenib was retrieved from PubChem (National Center for Biotechnology Information, PubChem Compound Summary for CID71657455, https://pubchem.ncbi.nlm.nih.gov/compound/Ivosidenib, accessed 6 October 2021). The chemical structure of LY3410738 is in the public domain^22^. Protonation states at pH 7.4 and tridimensional geometries were calculated by JChem (version 21.18, www.chemaxon.com). Molecular docking studies were run using the GOLD program (version 5.4, www.ccdc.cam.ac.uk). The binding region was defined as a 14-Å radius sphere centered on the center-of-mass of the co-crystalized ligand. The protein structures were considered rigid, with the exception of hydrogen-bonding functions and the entire side chains of IDH1 D/N279, IDH1 C269, and IDH1 W267. A slight constraint was applied to favor poses able to make electrostatic interactions with IDH1 D/N279 side chains. Non-covalent docking was performed for ivosidenib using otherwise default parameters. The adduct between IDH1 C269 and LY3410738 was constructed by modifying the hybridization of both acrylamide warhead carbons to sp3 as well as adding hydrogens and terminal bare sulfur. The latter atom and the sulfur of the IDH1 C269 side chain were defined for GOLD covalent docking using otherwise identical parameterization. The selected docking solutions, together with side chains presenting at least one atom at 4.5 Å, were subject to 100-step steepest-decent followed by 500-step conjugate-gradient minimization within the Amber Force Field. The resulting coordinates are the predicted binding modes of both inhibitors in the allosteric sites of both IDH1 mutants.

### Reporting summary

Further information on research design is available in the Nature Research Reporting Summary linked to this article.

## Supplementary information

Supplementary Figure 1

Reporting Summary

## Data Availability

Please see the “Methods” section.

## Code availability

No custom code or algorithms were created for this study.
