# Supplementary figures and images for: Secondary IDH1 resistance mutations and oncogenic IDH2 mutations cause acquired resistance to ivosidenib in cholangiocarcinoma
Source: NPJ Precis Oncol. 2022 Sep 2;6:61. doi: 10.1038/s41698-022-00304-5 (PMC9440204; doi:10.1038/s41698-022-00304-5)

**Supplementary Figure 1:** Uncropped and unprocessed scans (Figure 3a).

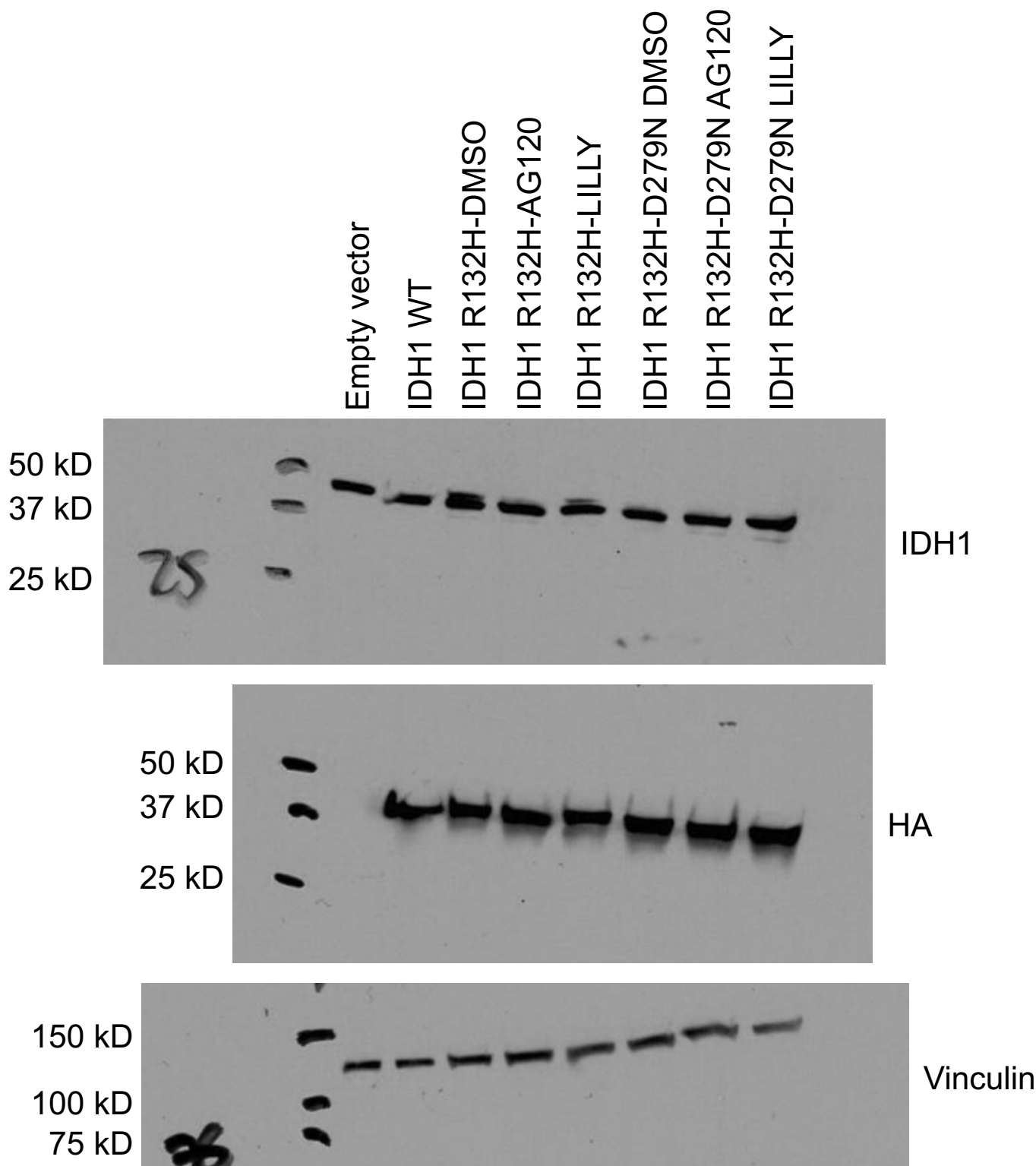

Supplement: Supplementary file 1 — Supplementary Figure 1 [file 41698_2022_304_MOESM1_ESM.pdf]
